# Effect of a 6-Month Controlled Lifestyle Intervention on Common Carotid Intima-Media Thickness

**DOI:** 10.1007/s12603-021-1628-0

**Published:** 2021-04-13

**Authors:** Christian Koeder, A. Hahn, H. Englert

**Affiliations:** 1 Department of Nutrition, University of Applied Sciences Münster, Corrensstr. 25, 48149, Münster, Germany; 2 Institute of Food Science and Human Nutrition, Leibniz University Hanover, Hanover, Germany

**Keywords:** Plant-based diet, healthy aging, preventive medicine, cardiovascular disease, cardiovascular health

## Abstract

**Objectives:**

The intima-media thickness of the common carotid artery (ccIMT) is an established risk marker for cardiovascular disease (CVD). However, it is unclear whether lifestyle interventions can easily demonstrate an improvement in ccIMT. The objective was to test if our intervention would beneficially affect ccIMT (among other CVD markers).

**Design:**

Non-randomized controlled trial

**Setting:**

Rural northwest Germany

**Participants:**

Middle-aged and elderly participants from the general population (intervention: n = 114; control: n = 87)

**Intervention:**

A community-based, 6-month controlled lifestyle intervention focusing on four areas of lifestyle change: a plant-based diet, physical activity, stress management, and an improved social life. A strong emphasis was on dietary change.

**Measurements:**

We tested whether ccIMT change from baseline to 6 months was different between groups.

**Results:**

With all participants included, no significant difference in mean ccIMT change between groups was observed (p = 0.708). However, in a subgroup analysis with participants with high baseline mean ccIMT (≥0.800 mm) a significant difference in mean ccIMT change between intervention (−0.023 [95% CI −0.052, 0.007] mm; n = 22; baseline mean ccIMT: 0.884 ± 0.015 mm) and control (0.041 [95% CI 0.009, 0.073] mm; n = 13; baseline mean ccIMT: 0.881 ± 0.022 mm) was observed (p = 0.004). Adjusting for potential confounders did not substantially alter the results.

**Conclusion:**

The results indicate that healthy lifestyle changes can beneficially affect ccIMT within 6 months and that such a beneficial effect may be more easily demonstrated if participants with high baseline ccIMT are recruited. The observed effect is of relevance for the prevention of CVD events, including myocardial infarction and stroke.

## Introduction

It is widely accepted that healthy dietary and lifestyle choices can lower CVD risk (1). Furthermore, pathological arterial wall changes that lead to CVD events may even be reversed (2, 3, 4). However, very few intervention studies have been successful in demonstrating such a reversal (5, 6), and some of these studies were not controlled (7, 8, 9, 10, 11), making their results less reliable. Parameters employed in studies to demonstrate the reversal of arterial wall pathology (atherosclerosis and smooth muscle growth) have been, for example, coronary artery occlusion (assessed by coronary arteriography (4, 5, 10)) and angina pectoris symptoms (9). A non-invasive parameter that could fulfil this task in non-symptomatic populations is ccIMT (12).

The parameter ccIMT is an established (albeit controversial) (13) marker of the progression of arterial wall pathology, subclinical organ damage, and the risk of future CVD events, including myocardial infarction and ischaemic stroke (12, 14). The measurement of ccIMT via ultrasound allows the assessment of pathological arterial wall changes while still at a subclinical stage (15).

A recent meta-analysis of intervention studies (including mostly pharmaceutical and dietary supplement trials) indicates that ccIMT change is a valid surrogate marker for CVD risk and as such a useful parameter for intervention studies (16).

The objective of the study was to test if our lifestyle intervention would lead to measurable improvements in ccIMT (among other CVD risk markers).

## Methods

### Participants

**Figure 1:**
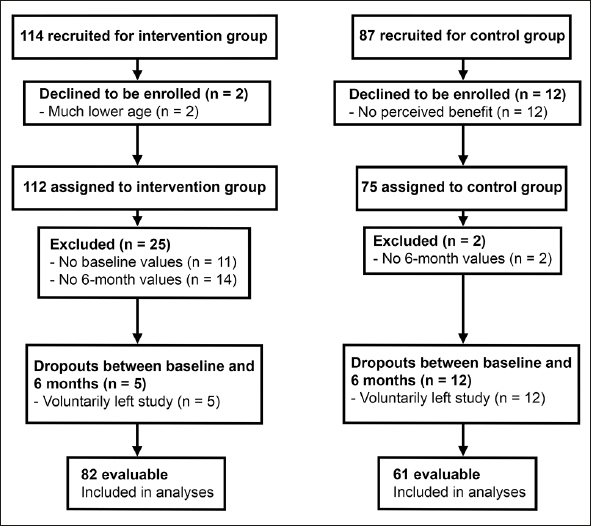
Flow chart of participants through the study

For the intervention and control groups 114 and 87 participants were recruited, respectively. The number of evaluable participants included in the analysis of ccIMT change was 82 in the intervention and 61 in the control group (Figure 1).

All subjects gave their written informed consent prior to inclusion in the study. The study was conducted in accordance with the Declaration of Helsinki, and the protocol was approved by the ethics committee of the Medical Association of Westphalia-Lippe and of the University of Münster (Münster, Germany; reference: 2018-171-f-S; approved 4 April 2018).

The intervention group was recruited, parameters were assessed, and the intervention was conducted in a small town in northwest Germany. The control group was recruited and assessments were made in another small town, nearby in the same region.

For the intervention group, participants were recruited from the general population via a health market (February 2018) and by word of mouth, while for the control group, participants were recruited at a local public event (September 2018). As the intervention was conducted as a community-based programme, subjects were not strictly preselected, but a similar age group was targeted, and a similar male-to-female ratio was aspired to in both groups. The only inclusion criteria were the physical and mental ability to take part in the study and to be ≥18 years of age.

### Study design

This controlled intervention study had a total duration of 18 months. Results of the first 6 months for the parameter ccIMT are presented in this article. The intervention consisted of a healthy lifestyle programme, while the control group received no intervention. The same parameters were assessed at equivalent time points in both groups: baseline, 10 weeks, 6, 12, and 18 months. The first 10 weeks of the intervention constituted the intensive part of the lifestyle programme. The parameter ccIMT was not assessed at 10 weeks as a significant change in such a short period of time was considered unlikely. Therefore, the first follow-up measurement of ccIMT was conducted at 6 months.

Participants were not randomized, and in lifestyle interventions, blinding participants to group allocation is not possible (17). Blinding of the ultrasound technician (who was assessing ccIMT) to group allocation was not feasible either. However, this technician was not involved in the implementation of any aspect of the intervention (17).

Both the intervention and control group study arms were conducted in parallel, but the control group study arm started and finished 6 months later than the intervention group (same duration of follow-up in both groups), as there were insufficient capacities to recruit and start both study arms at the same time.

### Study hypothesis

Healthy dietary patterns and other healthy lifestyle factors are associated with lower ccIMT values (18). In addition, some lifestyle interventions have demonstrated a reduction in ccIMT (19, 20, 21, 22, 23) or at least a slowed down increase in ccIMT (24, 25, 26). We therefore hypothesized that participants of our lifestyle intervention would demonstrate a significant decrease in ccIMT values from baseline to 6 months and that this decrease would be significantly larger than in the control group. The primary outcome measure of the study was body weight change and results regarding this and the remaining outcome parameters of the study will be published shortly.

### Lifestyle programme

The first 10 weeks (intensive phase) of the lifestyle intervention consisted of 14 consecutive seminars. The remainder of the intervention consisted of monthly seminars. Seminar topics focused on a healthy plant-based diet (27), healthy levels of physical activity, management of psychological stress, community support, and self-motivation, with a strong emphasis on dietary change. The seminars included short practical units such as cookery demonstrations or sessions with invited guests, including local general practitioners. Participants were given the opportunity to take part in eight additional workshops in smaller groups (∼20 participants each; ∼1-hour duration) which included cookery classes, a guided shopping tour, archery and table tennis workshops, and a relaxation workshop in nature. Dietary recommendations were to move towards a healthy, plant-based diet, i.e. to consume more healthy plant-based foods (fruit, vegetables, legumes including soya foods, whole grains, nuts, seeds, and healthy oils) and to consume less meat, butter, full-fat dairy, eggs, salt, added sugars, and highly processed foods and to avoid alcohol excess. Critical nutrients in plant-based diets, including vitamin B12, vitamin D, calcium, and iodine were discussed and adequate sources were communicated. No strict dietary rules or limits on portion sizes were given. Plant-based diets were defined as being predominantly based on plant foods. Such dietary patterns can be non-vegetarian, or they could be vegetarian or vegan as well. Dietary recommendations were monitored with semi-quantitative food logs. Apart from the seminars and workshops, participants received a healthy lifestyle handbook, a recipe booklet, a laminated one-page sheet with an overview of the lifestyle recommendations, and (after the intensive first 10 weeks) a monthly e-mail newsletter. The intervention group also received two one-on-one health coaching sessions (∼15 min each), one at baseline and one at 10 weeks (before and after the intensive phase).

### Carotid ultrasonography

Measurements of ccIMT were conducted following a strict protocol in accordance with the Mannheim consensus (28). Only the far wall was scanned. Measurements were taken at the time of the widest luminal distention during the cardiac cycle (29, 30). All measurements were made with the same ultrasound device (Mindray DC-N3, Mindray, Shenzhen, China), equipped with a high-resolution linear array transducer and automated digital edge detection software (Auto IMT). A frequency of 8.5 MHz and an image depth of 37 mm were used. Zoom was not used (31). The precision, provided by the manufacturer with which the intima-media thickness could be assessed was 0.01 mm. Within each 1 cm segment, the software automatically measured the intima-media thickness at 149 measurement point pairs and from these computed the mean (mean ccIMT) and maximal (max ccIMT) values. Two measurements were taken on each side. These four measurements resulted in four mean and four max values per person, per measurement time point. The data presented here are the (group) means of the (individual) means of each of these four mean values (mean ccIMT) or max values (max ccIMT), respectively. All measurements were made by the same technician, an internist with previous experience in ccIMT measurement with this device.

### Statistical analyses

A sample size calculation was performed based on the primary outcome measure of the study, which was change in body weight. This calculation was based on data from a pilot study with a prototype version of the lifestyle programme (32). Assuming a dropout rate of at least 10%, a minimum sample size of 93 participants (intervention: 62; control: 31) was indicated to reach a global power of 0.8 and a global significance level of 0.05.

For the secondary end point of mean ccIMT change (from baseline to 6 months) an additional sample size calculation was performed using data from comparable studies (23, 26). Based on our expectation of a change in mean ccIMT of −0.100 mm from baseline to 6 months in the intervention group (effect size: ∼0.5) (23), and no change in the control group, our actual sample size was adequate to detect a difference in mean ccIMT with a power of 0.8 and at a significance level of 0.05. Any detected differences in secondary end points, including ccIMT, are considered exploratory. Initially, actual sample size was moderately higher than required (intervention: n = 114; control: n = 87; Figure 1).

Shapiro-Wilk test was used to assess the data for non-normality, and p <0.05 was defined as describing a non-normal distribution. For comparing baseline characteristics between groups, Fisher's exact test was used for categorical variables, while independent t-test was used for normally distributed and Mann-Whitney U test for non-normally distributed continuous variables (all tests were two-sided). To evaluate within-group mean ccIMT and max ccIMT changes, in the intervention and control groups, respectively, paired t-test was used for normally distributed and Wilcoxon test was used for non-normally distributed data (all tests were two-sided).

To evaluate the difference in ccIMT change (mean and max, respectively) between the two groups a one-way analysis of covariance (ANCOVA) was used, using the baseline ccIMT values (mean and max, respectively) as covariates (33, 34). The ANCOVA analyses were then repeated adjusting for several confounders gained from the literature [35, 36, 37, 38] and from associations observed in this study population. Apart from baseline mean or max ccIMT, the covariates adjusted for were sex, age, smoker status, body mass index (BMI), total cholesterol, LDL cholesterol, HDL cholesterol, systolic and diastolic blood pressure, and HbA1c. ANOVA has been shown to be robust against non-normally distributed data [39]. Furthermore, exploratory subgroup analyses were conducted including only participants with baseline mean ccIMT ≥0.800 mm. In sensitivity analysis, results were additionally adjusted for changes in medication (blood pressure, diabetes, and cholesterol-lowering medication). In further sensitivity analyses, instead of adjusting for baseline values, we adjusted for the mean of the baseline and 6-month values (33, 40).

Correlations of baseline ccIMT with other CVD risk markers were assessed with Spearman's rho correlations (two-sided). All blood parameters, vital parameters, and anthropometric measurements were assessed in the fasted state. In addition, to determine the repeatability (within-assay precision) of repeated left and right measurements of ccIMT at one time point, Spearman's rho correlations (two-sided) were calculated.

All analyses were based on unimputed data (complete case analysis). Blinding was not feasible for statistical analysis. The analysis strategy was intention to treat (41). Statistical significance was consistently set at the 0.05 level. All analyses were conducted using IBM SPSS Statistics (Version 25.0. Armonk, NY). Baseline values are given as mean ± standard error of the mean (SEM).

## Results

### Compliance

In the intervention group, compliance, as defined by seminar attendance during the 10-week intensive phase of the lifestyle programme, was relatively high, with 61 out of the 82 evaluable participants (74.4%) attending ≥11 (out of 14) seminars.

### Baseline characteristics

For a total of 143 participants (intervention: 82; control: 61) ccIMT values were available for both measurement time points (baseline and 6 months), and these participants were included in the analysis. The flow of participants through the study is shown in Figure 1.

The distribution of male and female participants was not significantly different between groups (p = 0.378). Mean age was higher in the intervention than in the control group (p = 0.005; Table 1). Baseline mean ccIMT and max ccIMT, systolic and diastolic blood pressure, body weight, BMI, and waist circumference were not significantly different between the two groups (Table 1). Equally, there were no statistically significant differences between the two groups in terms of baseline cholesterol (total, LDL, and HDL), triglycerides, fasting glucose, HbA1c, fasting insulin, or resting heart rate (unpublished results). In addition, the two groups did not differ in the distribution of their smoker status (p = 0.105) or marital status (p = 0.952; Table 1). Furthermore, there were no significant differences between the two groups in terms of alcohol intake frequency or the percentage of participants with any of a variety of diagnosed disease conditions assessed (hypertension, dyslipidaemia, heart disease, peripheral artery disease, diabetes, retinopathy, peripheral neuropathy, diabetic foot, kidney disease, allergies, gastrointestinal disease, thyroid disease, depression, rheumatoid arthritis, chronic pain, lung disease, bone disease as well as “other disease” or “free of diagnosed disease”). In addition, there were no significant differences between the two groups in terms of the percentage of participants with a history of stroke, a history of cancer, a family history (siblings, parents, grandparents) of myocardial infarction or stroke, or the percentage of participants who, based on baseline values, had hypertension, high total cholesterol, high LDL cholesterol, low HDL cholesterol, or high triglycerides. However, the percentage of participants with baseline HbAlc ≥6.5% was higher in the control group (4 individuals) than in the intervention group (0 individuals; p = 0.032; Supplementary table 1).

**Table 1:** Baseline characteristics of evaluable participants

**Variable**	**Intervention group (n = 82)**	**Control group (n = 61)**	**p-value #**
Men, n (%)	26 (31.7)	24 (39.3)	0.378^a^
Age at baseline, years	59.4 ± 1.0	54.7 ± 1.4	0.005^b^
Body weight, kg	81.9 ± 2.0	85.2 ± 2.5	0.244^c^
BMI, kg/m^2^	27.7 ± 0.6	28.3 ± 0.8	0.665^c^
Waist circumference, cm	98.8 ± 1.6	97.8 ± 2.0	0.777^c^
Systolic BP, mm Hg	133.3 ± 1.7	131.8 ± 2.1	0.568^b^
Diastolic BP, mm Hg	80.3 ± 0.9	79.4 ± 1.3	0.648^c^
Mean ccIMT, mm	0.698 ± 0.015	0.672 ± 0.018	0.277^b^
Max ccIMT, mm	0.863 ± 0.018	0.823 ± 0.022	0.130^c^
Smoker status, n (%)	Current/occasional: 8 (9.8) Ex: 27 (32.9) Never: 47 (57.3)	Current/occasional: 14 (23.0) Ex: 17 (27.9) Never: 30 (49.2)	0.105^a^
Marital status, n (%)	Married: 69 (84.1) Partner, unmarried: 4 (4.9) Single (not widowed): 6 (7.3) Single (widowed): 3 (3.7) Missing data: 0 (0.0)	Married: 51 (83.6) Partner, unmarried: 3 (4.9) Single (not widowed): 3 (4.9) Single (widowed): 3 (4.9) Missing data: 1 (1.6)	0.952^a^


Values are means ± SEM except for qualitative variables, expressed as n (%); BMI: body mass index; BP: blood pressure; ccIMT: common carotid intima-media thickness; SEM: standard error of the mean; # p-value for comparisons between groups by: a. Fisher's exact test (two-sided); b. independent t-test (two-sided); c. Mann-Whitney U test (two-sided)

### Baseline mean and max ccIMT stratified by risk factors

At baseline, for the overall study population (n = 143) mean ccIMT was 0.687 ± 0.012 mm, and max ccIMT was 0.846 ± 0.014 mm.

At baseline, mean ccIMT values were significantly higher in the left (0.701 ± 0.014 mm) compared to the right (0.677 ± 0.013 mm) carotid artery (n = 124; p = 0.009). Similarly, at baseline max ccIMT values were significantly higher on the left (0.857 ± 0.016 mm) compared to the right (0.824 ± 0.016 mm) side (n = 124; p = 0.015).

At baseline, men (n = 50) had higher ccIMT values than women (n = 93) (mean ccIMT: p = 0.005; max ccIMT: p = 0.011).

### Bivariate correlations of ccIMT with other CVD risk factors

Combining participants of both the intervention and control groups, baseline mean ccIMT did not significantly correlate with baseline total cholesterol, LDL cholesterol, HDL cholesterol, triglycerides, insulin, diastolic blood pressure, resting heart rate, body height, body weight, or BMI. However, baseline mean ccIMT positively correlated with glucose (r = 0.304; p <0.001), HbA1c (r = 0.238; p = 0.004), systolic blood pressure (r = 0.309; p <0.001), waist circumference (r = 0.207; p = 0.013), and age (r = 0.618; p <0.001).

Out of the above-mentioned parameters, baseline max ccIMT positively correlated with glucose (r = 0.351; p <0.001), HbA1c (r = 0.285; p = 0.001), insulin (r = 0.184; p = 0.028), systolic blood pressure (r = 0.313; p <0.001), waist circumference (r = 0.296; p <0.001), BMI (r = 0.174; p = 0.037), and age (r = 0.584; p <0.001).

Combining participants of both the intervention and control groups, mean ccIMT change (progression from baseline to 6 months) did not significantly correlate with the progression values of cholesterol (total, LDL, or HDL), triglycerides, glucose, HbA1c, insulin, systolic or diastolic blood pressure, resting heart rate, body weight, waist circumference, or BMI. However, mean ccIMT change negatively correlated with baseline mean ccIMT (r = −0.272; p = 0.001).

Similarly, max ccIMT change did not correlate with any of the above-mentioned progression values, but max ccIMT progression negatively correlated with baseline max ccIMT (r = −0.317; p <0.001).

### Repeatability

Repeatability (within-assay precision) of mean ccIMT and max ccIMT measurements at one time point, on the left and right side, respectively, was generally good (r ≥0.94 for mean ccIMT, and r ≥0.90 for max ccIMT).

Mean differences in repeated measurements were small for mean ccIMT (baseline left: 0.003 mm, n = 110; baseline right: 0.004 mm, n = 105; 6 months left: 0.002 mm, n = 131; 6 months right: <0.001 mm, n = 134), as well as for max ccIMT (baseline left: 0.007 mm, n = 110; baseline right: 0.002 mm, n = 105; 6 months left: 0.005 mm, n = 131; 6 months right: 0.003 mm, n = 134; occasionally there were individual missing values due to low image quality, usually related to anatomical factors, especially high body fat.)

### Mean and max ccIMT change from baseline to 6 months

From baseline to 6 months, mean ccIMT significantly increased by 0.018 (95% CI 0.003, 0.032) mm in the intervention group (p = 0.005) and significantly increased by 0.025 (95% CI 0.010, 0.039) mm in the control group (p = 0.001). The difference between these two changes was not statistically significant (p = 0.708; adjusted for mean ccIMT baseline values).

From baseline to 6 months, max ccIMT non-significantly increased in both groups (intervention: p = 0.126; control: p = 0.133), and there was no significant difference between the two groups (p = 0.928; adjusted for max ccIMT baseline values; Table 2).

**Table 2:** Changes in mean ccIMT and max ccIMT from baseline to 6 months

**Variable**	**Intervention group (n = 82)**	**Control group (n = 61)**	**p-value #**	**p-value # (multivariable- adjusted)**
Change in mean ccIMT (mm)	0.018 (95% CI 0.003, 0.032)	0.025 (95% CI 0.010, 0.039)	0.708^a^	0.835^b^
Change in max ccIMT (mm)	0.007 (95% CI −0.016, 0.029)	0.014 (95% CI −0.006, 0.035)	0.928^c^	0.848^d^


Values are means and 95% confidence intervals; ANCOVA: one-way analysis of covariance; BMI: body mass index; ccIMT: common carotid intima-media thickness.; # p-value for comparisons between groups by: a. ANCOVA, adjusted for baseline mean ccIMT; b. ANCOVA, adjusted for baseline mean ccIMT, sex, age, smoker status, BMI, total cholesterol, LDL cholesterol, HDL cholesterol, systolic and diastolic blood pressure, and HbA1c (intervention group: n = 81; control group: n = 61); c. ANCOVA, adjusted for baseline max ccIMT; d. ANCOVA, adjusted for baseline max ccIMT, sex, age, smoker status, BMI, total cholesterol, LDL cholesterol, HDL cholesterol, systolic and diastolic blood pressure, and HbA1c (intervention group: n = 81; control group: n = 61)

In a subgroup analysis including only the participants with baseline mean ccIMT ≥0.800 mm, in the intervention group (n = 22) there was a non-significant decrease in mean ccIMT of ≥0.023 (95% CI ≥0.052, 0.007) mm from baseline to 6 months (p = 0.205) while in the control group (n = 13) there was a significant increase in mean ccIMT of 0.041 (95% CI 0.009, 0.073) mm (p = 0.017).

The difference between these two changes was statistically significant (p = 0.004; adjusted for mean ccIMT baseline values; Table 3), and this constituted a between-group difference of 0.063 (95% CI 0.020, 0.107) mm.

**Table 3:** Subgroup analysis: changes in mean ccIMT and max ccIMT from baseline to 6 months in participants with baseline mean ccIMT ≥0.800 mm

**Variable**	**Intervention group (n = 22)**	**Control group (n = 13)**	**p-value #**	**p-value # (multivariable- adjusted)**
Change in mean ccIMT, mm	−0.023 (95% CI −0.052, 0.007)	0.041 (95% CI 0.009, 0.073)	0.004^a^	0.004^b^
Change in max ccIMT, mm	−0.034 (95% CI −0.067, −0.001)	0.025 (95% CI −0.029, 0.079)	0.020^c^	0.019^d^


Values are means and 95% confidence intervals; ANCOVA: one-way analysis of covariance; ccIMT: common carotid intima-media thickness. # p-value for comparisons between groups by: a. ANCOVA, adjusted for baseline mean ccIMT; b. ANCOVA, adjusted for baseline mean ccIMT, sex, and age; c. ANCOVA, adjusted for baseline max ccIMT; d. ANCOVA, adjusted for baseline max ccIMT, sex, and age

This difference remained statistically significant after adjusting for baseline mean ccIMT, sex, and age (p = 0.004). Due to the low number of cases only these three covariates were adjusted for, but in a sensitivity analysis, additionally adjusting for smoker status, BMI, total cholesterol, LDL cholesterol, HDL cholesterol, systolic and diastolic blood pressure, and HbA1c did not change the result (p = 0.029). This result also remained significant after adjusting for baseline mean ccIMT, sex, age, and smoker status as well as for progression values (changes from baseline to 6 months) of BMI, total cholesterol, LDL cholesterol, HDL cholesterol, systolic and diastolic blood pressure, and HbA1c (p = 0.037; sensitivity analysis). Furthermore, this result remained significant after adjusting for baseline mean ccIMT, sex, age, and changes in medication (p = 0.009; sensitivity analysis).

In a further sensitivity analysis, instead of adjusting for baseline mean ccIMT, we adjusted for the mean of the baseline and 6-month mean ccIMT values. This did not substantially change the result (p = 0.008).

In this same subgroup (participants with a baseline mean ccIMT ≥0.800 mm), in the intervention group (n = 22) there was a decrease in max ccIMT of -0.034 (95% CI −0.067, −0.001) mm from baseline to 6 months (p = 0.047) while in the control group (n = 13) there was a non-significant increase in max ccIMT of 0.025 (95% CI −0.029, 0.079) mm (p = 0.333).

The difference between these two changes was statistically significant (p = 0.020; adjusted for max ccIMT baseline values; Table 3). This constituted a between-group difference of 0.059 (95% CI 0.001, 0.116) mm.

This difference remained statistically significant after adjusting for baseline max ccIMT, sex, and age (p = 0.019). In a sensitivity analysis, additionally adjusting for smoker status, BMI, total cholesterol, LDL cholesterol, HDL cholesterol, systolic and diastolic blood pressure, and HbA1c did not change the result (p = 0.042). Furthermore, this result remained significant after adjusting for baseline max ccIMT, sex, age, and medication change (p = 0.036; sensitivity analysis). This result was attenuated when adjusting for baseline max ccIMT, sex, age, and smoker status as well as for progression values of BMI, cholesterol (total, LDL, and HDL), blood pressure (systolic and diastolic), and HbA1c (p = 0.121; sensitivity analysis). However, except for sex, none of the covariates in the ANCOVA model had a significant influence on the model.

In a further sensitivity analysis, instead of adjusting for baseline max ccIMT, results were adjusted for the mean of the baseline and 6-month max ccIMT values. This also attenuated the results for max ccIMT change (p = 0.108) However, this covariate had no significant influence on the model.

### Adverse events

No adverse events related to the study were observed in either study group.

## Discussion

Contrary to our study hypothesis, with all participants included, mean ccIMT did not decrease in the intervention group but rather increased in both the intervention and control groups, highlighting the importance of a control group. Also contrary to our hypothesis, with all participants included, our results showed no significant difference in mean ccIMT change between intervention and control. This indicates that the short-term effect of lifestyle on ccIMT may not be detectable in non-symptomatic individuals from the general population without increased baseline ccIMT values. However, in subgroup analyses of participants with baseline mean ccIMT of ≥0.800 mm a significant difference in mean ccIMT change was observed between intervention and control, favouring the intervention group. This difference between intervention and control remained significant after multivariable adjustment, which suggests that it might have been a beneficial result of the intervention programme. This difference also remained significant when adjusting for progression values of several common CVD risk markers (BMI, cholesterol levels, blood pressure, and HbA1c), which indicates that this observed treatment effect was independent of changes in BMI (42), cholesterol (43), blood pressure (44), and long-term blood glucose levels (45), which are generally assumed to be major determinants of ccIMT. This suggests that, as with CVD risk in general (46), there are other factors that influence ccIMT apart from the classic CVD risk markers and that at least some of these factors can also be influenced by diet and other lifestyle factors.

In our subgroup analysis with participants with baseline mean ccIMT ≥0.800 mm the difference in mean ccIMT change between the intervention and control group was 0.063 (95% CI 0.020, 0.107) mm. This indicates that the observed difference may be clinically relevant. Willeit et al. (2020), in a large-scale meta-analysis of intervention studies, have shown that each 0.010 mm/year slower progression of ccIMT reduced the risk of myocardial infarction by 12% and of stroke by 8% (mean follow-up: 3.7 years), with the subgroup of dietary interventions showing more consistent results than the medication-based interventions (16).

In a prospective cohort study from Italy with a follow-up of 12 years, Olmastroni et al. (2019) showed that both mean and max ccIMT increase more rapidly with age in individuals who develop multifocal carotid atherosclerosis, which indicates that long-term ccIMT change is a marker of atherosclerosis development (36). It can therefore be hypothesized that significant differences in short-term ccIMT change between intervention and control, as seen in our subgroup, reflect real differences in pathological arterial wall changes.

The question remains why in our study, with all participants included, there was an increase in mean ccIMT in both the intervention (mean: 0.017 mm) and control groups (mean: 0.025 mm; Table 2) in only 6 months. In a large international analysis of 31 cohorts, Lorenz et al. (2018) reported a mean annualised mean ccIMT change of 0.01 mm, with a range of −0.10 to 0.05 mm (35), and our results fall within this range. This large variability in ccIMT change in different studies is possibly due to a combination of measurement error, random fluctuations (that cannot be controlled), interindividual differences, and actual ccIMT changes being relatively small and nonlinear (35, 36).

### Comparison with results from the literature

In other studies, associations between mean ccIMT and a variety of non-classic CVD markers in blood have been observed, including a positive correlation with high-sensitivity C-reactive protein (hs-CRP) (47), which we also assessed in our study. However, in the subgroup of participants with baseline mean ccIMT ≥0.800 mm no significant difference in hs-CRP changes was shown between intervention and control (unpublished results), which indicates that the observed difference in ccIMT change was unrelated to hs-CRP.

Similarly to our results, in the PREDIMED-Navarra study, a randomized controlled trial from Spain using a traditional Mediterranean diet with either virgin olive oil or nuts as the intervention, with all participants included, after 1 year no significant difference in mean ccIMT change was found between groups (48). However, in subgroup analyses including only participants with baseline mean ccIMT ≥0.9 mm, both in the olive oil and the nut intervention groups a significant mean ccIMT decrease of −0.093 mm and −0.086 mm, respectively, was observed, with no significant change in the control group (all three groups combined: n = 61) (48). In the analysis adjusted for age, sex, and hyperlipidaemia at baseline, the differences in mean ccIMT change between the intervention and control group were statistically significant (48). Like the PREDIMED-Navarra study, our results indicate that mean ccIMT may favourably respond to healthy lifestyle changes only (or more strongly so) in subjects with more advanced unfavourable arterial wall changes.

The majority of controlled lifestyle (including diet and/or exercise) interventions have failed to demonstrate a clear effect on ccIMT compared to control (24, 49), while only one of these unsuccessful studies reported a subgroup analysis with high-risk participants (50). However, some controlled trials using diet and/or exercise have been able to show a favourable effect on ccIMT (decrease or slowed progression), and when dietary recommendations were given, these included advice to adhere to a traditional Mediterranean diet (which is a plant-based dietary pattern) (51, 52) or to consume less salt and alcohol and more fruit and vegetables (53), and in one study, more dairy (20) (all randomized controlled trials).

In observational studies, a healthier, more plant-based diet (more fruit, vegetables, legumes, and whole grains, moderate alcohol intake, and less red meat) has been associated with more favourable mean ccIMT changes over time (54). In contrast, one study from Spain did not find an association between diet quality and ccIMT, but in terms of diet quality, vegetable oils (other than olive and sunflower oil) were rated unfavourable, breakfast flakes and daily alcohol consumption were rated favourable, and no differentiation was made between refined and whole grains (55).

### Strengths and weaknesses

A strength of our study is the use of a strict standardized measurement protocol and the averaging of four measurements per person per time point. Moreover, all ultrasound scans were conducted by the same technician (and with the same device), which excludes confounding due to inter-operator variability (13). In addition, repeatability of ultrasound measurements was high and comparable to previous studies (36, 56).

Our study had several weaknesses: first, while ccIMT was intentionally not assessed at 10 weeks (the end of the intensive phase of the intervention programme), but only at 6 months, we did observe that cholesterol levels in the intervention group decreased from baseline to 10 weeks but increased again from 10 weeks to 6 months (unpublished results). This development likely influenced ccIMT and this influence could not be examined. Second, ccIMT was measured at the widest luminal distention, which is comparable to peak-systole measurements (when ccIMT is slightly smaller), while it is more common to assess ccIMT at end-diastole (when ccIMT is slightly larger) (29). However, both approaches are reliable if consistently used (29). Third, the control group started with a delay of 6 months (same follow-up duration) compared to the intervention group. Seasonal variations in vitamin D status, for example, could influence ccIMT, but this effect is uncertain (57). An analysis of all time points of the study, which will also include dietary intake, will be published shortly.

Due to the community-based nature of our study, participants were not randomized individually as participants of the control group were not supposed to be aware of the contents of the intervention (58). This was achieved by recruiting the intervention and control groups in two separate small towns. Cluster randomization of the two study centres was not conducted, as a large volume of preparatory work was necessary in the intervention community before the beginning of the study (obtaining support from the mayor, finding adequate premises for events, involving local general practitioners, health workers, and the local press). Cluster randomization would have meant making these preparations in two municipalities for which there was insufficient time, as the funding was received at short notice and for an immediate, specific time period. This would also have necessitated withholding all information about the interventions' contents from local stakeholders in both municipalities and then informing one group of stakeholders that their municipality had not been chosen because of randomization. Such an approach was not considered feasible or ethical and could have ruined our reputation and endangered compliance. Furthermore, it has been shown that any benefits of randomization to protect against selection bias in health care trials are uncertain (59).

### Future research

Future controlled trials assessing ccIMT progression should report both the within-group effects as well as a between-group comparison. Uncontrolled studies should not be undertaken, and there should always be a control group which does not receive an intervention. The control group at baseline should be comparable to the intervention group. Journal articles should always clearly state which section of the carotid artery was assessed (common, internal, bifurcation, or a combination thereof). Dietary recommendations given should be well-designed (1). Well-designed dietary and lifestyle interventions assessing ccIMT progression, with participants with high baseline ccIMT values (such as mean ccIMT ≥0.800 mm), may be able to demonstrate a direct beneficial effect of healthy lifestyle changes on artery health and could as such facilitate and speed up much-needed changes in the health care system (16).

## Conclusion

Our study failed to confirm our hypothesis that the participants of our lifestyle programme (recruited from the general population in rural northwest Germany) would show a significant decrease in ccIMT after 6 months and that this decrease would be significantly more favourable than in the control group. However, in a subgroup analysis of participants with baseline mean ccIMT ≥0.800 mm we observed a significant and clinically relevant difference in ccIMT change between intervention and control, favouring the intervention group. Our results indicate that mean ccIMT change can be a suitable CVD risk parameter for lifestyle intervention studies (60) if individuals with high baseline ccIMT can be included. We would like to encourage other working groups assessing mean ccIMT progression to conduct subgroup analyses with a cut-off value for high baseline ccIMT, as this could corroborate or contradict our conclusions.

## Trial registration

German Clinical Trials Register DRKS (reference: DRKS00018775; www.drks.de).

## Copyright comment

corected publication in 2021
