# Supplementary material for: Effect of a 6-Month Controlled Lifestyle Intervention on Common Carotid Intima-Media Thickness
Source: J Nutr Health Aging. 2021 Apr 13;25(7):869–77. doi: 10.1007/s12603-021-1628-0 (PMC12280572; doi:10.1007/s12603-021-1628-0)
Supplement: Supplementary file 1 — Appendix [file mmc1.docx]

| **Supplementary table 1.** Additional baseline characteristics of evaluable participants | | | |
| --- | --- | --- | --- |
| **Variable** | **Intervention group**  **(n = 82)** | **Control group**  **(n = 61)** | **p-value ^#^** |
| Diagnosed hypertension, n (%) | 31 (37.8)  [n = 81] | 23 (37.7)  [n = 60] | 1.000 |
| Hypertension based on baseline values, n (%) § | 54 (65.9) | 38 (62.3) | 0.725 |
| Diagnosed dyslipidaemia, n (%) | 4 (4.9)  [n = 81] | 1 (1.6)  [n = 60] | 0.394 |
| Total cholesterol ≥200 mg/dl at baseline, n (%) | 45 (54.9)  [n = 81] | 36 (59.0) | 0.734 |
| High LDL cholesterol (measured) ≥130 mg/dl at baseline, n (%) | 42 (51.2)  [n = 81] | 36 (59.0) | 0.496 |
| LDL cholesterol (calculated) ≥130 mg/dl at baseline, n (%) | 32 (39.0)  [n = 81] | 27 (44.3)  [n = 60] | 0.605 |
| HDL cholesterol <40 mg/dl at baseline, n (%) | 4 (4.9)  [n = 81] | 5 (8.2) | 0.498 |
| Triglycerides ≥150 mg/dl at baseline, n (%) | 15 (18.3)  [n = 81] | 10 (16.4) | 0.826 |
| Family history of heart attack or stroke (siblings), n (%) | 6 (7.3)  [n = 81] | 5 (8.2)  [n = 59] | 1.000 |
| Family history of heart attack or stroke (parents), n (%) | 44 (53.7)  [n = 81] | 24 (39.3)  [n = 60] | 0.125 |
| Family history of heart attack or stroke (grandparents), n (%) | 20 (24.4)  [n = 77] | 8 (13.1)  [n = 56] | 0.132 |
| Diagnosed heart disease, n (%) | 7 (8.5)  [n = 81] | 7 (11.5)  [n = 60] | 0.580 |
| History of stroke, n (%) | 1 (1.2)  [n = 81] | 3 (4.9)  [n = 60] | 0.312 |
| Diagnosed peripheral artery disease, n (%) | 1 (1.2)  [n = 81] | 0  [n = 60] | 1.000 |
| Diagnosed diabetes, n (%) | 5 (6.1)  [n = 81] | 4 (6.6)  [n = 60] | 1.000 |
| Diagnosed retinopathy, n (%) | 3 (3.7) [n = 81] | 3 (4.9) [n = 60] | 0.699 |
| Diagnosed peripheral neuropathy, n (%) | 5 (6.1) [n = 81] | 3 (4.9) [n = 60] | 1.000 |
| Diagnosed diabetic foot, n (%) | 1 (1.2)  [n = 81] | 1 (1.6)  [n = 60] | 1.000 |
| HbA1c ≥6.5% at baseline, n (%) | 0  [n = 81] | 4 (6.6) | **0.032** |
| Diagnosed kidney disease, n | 0  [n = 81] | 0  [n = 60] | - |
| Diagnosed allergy, n (%) | 2 (2.4)  [n = 81] | 1 (1.6)  [n = 60] | 1.000 |
| Diagnosed gastrointestinal disease, n (%) | 3 (3.7)  [n = 81] | 2 (3.3)  [n = 60] | 1.000 |
| Diagnosed thyroid disease, n (%) | 10 (12.2)  [n = 81] | 3 (4.9)  [n = 60] | 0.155 |
| Diagnosed depression, n (%) | 2 (2.4)  [n = 81] | 3 (4.9)  [n = 60] | 0.651 |
| History of cancer, n (%) | 7 (8.5)  [n = 81] | 1 (1.6)  [n = 60] | 0.138 |
| Diagnosed rheumatoid arthritis, n (%) | 4 (4.9)  [n = 81] | 7 (11.5)  [n = 60] | 0.204 |
| Diagnosed chronic pain, n (%) | 9 (11.0)  [n = 81] | 7 (11.5)  [n = 60] | 1.000 |
| Diagnosed lung disease, n (%) | 9 (11.0)  [n = 81] | 7 (11.5)  [n = 60] | 1.000 |
| Diagnosed bone disease, n (%) | 15 (18.3)  [n = 81] | 16 (26.2)  [n = 60] | 0.305 |
| Other diagnosed disease, n (%) | 17 (20.7)  [n = 81] | 12 (19.7)  [n = 60] | 1.000 |
| Free of diagnosed disease, n (%) | 18 (29.0)  [n = 81] | 16 (26.2)  [n = 60] | 0.557 |
| Alcohol intake frequency, categories (questionnaire), n (%) | - Never: 1 (1.2) - Once per month or less often: 16 (19.5) - 2–4 times/month: 33 (40.2) - 2–3 times/week: 20 (24.4) - ≥4 times/week: 12 (14.6) | - Never: 0 - Once per month or less often: 13 (21.3) - 2–4 times/month: 28 (45.9) - 2–3 times/week: 15 (24.6) - ≥4 times/week: 4 (6.6)   [n = 60] | 0.566 |
| Values are numbers of individuals (percentage) in the study population.  § Hypertension: systolic blood pressure ≥130 and/or diastolic blood pressure ≥80 mmHg;  # p-value for comparisons between groups by Fisher’s exact test (two-sided) | | | |
